# Removal of the Tumor Thrombus from the Right Atrium without Extracorporeal Circulation: Emphasis on the Displacement of the Tumor Apex

**DOI:** 10.1155/2020/6063018

**Published:** 2020-06-18

**Authors:** D. V. Shchukin, V. N. Lesovoy, G. G. Khareba, A. I. Harahatyi, A. V. Maltsev, M. M. Polyakov, R. V. Stetsyshyn, M. P. Kopytsya, P. V. Mozzhakov, O. O. Makovozov

**Affiliations:** ^1^Kharkiv National Medical University, Kharkiv 61022, Ukraine; ^2^V.I. Shapoval Regional Clinical Center of Urology and Nephrology, Kharkiv 61037, Ukraine; ^3^Kharkiv Medical Academy of Postgraduate Education, Kharkiv 61176, Ukraine; ^4^L.T. Malaya Therapy National Institute of the National Academy of Medical Sciences of Ukraine, Kharkiv 61039, Ukraine; ^5^Dniprovsky Regional Clinical Oncology Dispensary, Dnipro 49055, Ukraine

## Abstract

**Objectives:**

To assess the outcomes of cavoatrial tumor thrombus removal using the liver transplantation technique for thrombectomy, a retrospective study was conducted.

**Materials and Methods:**

Five patients with atrial tumor thrombi who underwent piggy-back mobilization of the liver, surgical access to the right atrium from the abdominal cavity, and external manual repositioning of the thrombus apex below the diaphragm (milking maneuver) were included into the study. Extracorporeal circulation was used in none of the cases. The average length of the atrial component of the tumor was 20.0 ± 11.7 mm (10 to 35 mm), and the width was 14.8 ± 8.5 mm (10 to 30 mm). In this work, the features of patients and surgical interventions as well as perioperative complications and mortality were analyzed.

**Results:**

External manual repositioning of the tumor thrombus apex below the diaphragm was successfully performed in all patients. Tumor thrombi with the length of the atrial part up to 1.5 cm were removed through the extrapericardial approach. For evacuation of the thrombi with the large atrial part (3.0 cm or more), a transpericardial surgical approach was required. Specific complications associated with the access to the right atrium from the abdominal cavity (paresis of the right phrenic nerve, pneumothorax, and mediastinitis) were not detected in any case. The average clamping time of the supradiaphragmatic inferior vena cava (IVC) was 6.3 ± 4.6 min. The volume of intraoperative blood loss varied from 2500 to 5600 ml (an average of 3675 ± 1398.5 ml).

**Conclusion:**

Our work represents the initial experience in the liver transplantation technique for thrombectomy in distinct and well-selected patients with atrial tumor thrombi. The effectiveness of this approach needs further study. The video presentation of our research took place in March 2019 at the 34th Annual EAU Congress in Barcelona.

## 1. Introduction

One of the most important aspects of surgical treatment for renal tumors extending into the inferior vena cava (IVC) is control of the apical part of the tumor thrombus. This stage can be quite challenging in case of “high” localization of the thrombus apex (retrohepatic or intrapericardial IVC and right atrium) and mainly depends on the type of surgical approach selected. Traditionally, for these patients, cardiopulmonary bypass with or without deep hypothermic circulatory arrest is used [1].

However, this surgical technique results in wide opening of several body cavities, significantly increases the duration of surgery, and also can be accompanied by specific postoperative complications such as mediastinitis, sternal pain syndrome, pericardial adhesion scars, coagulopathy, and central nervous system complications [2, 3].

In recent years, there have been more and more reports on alternative surgical approaches without use of cardiopulmonary bypass and circulatory arrest in patients with the tumor thrombi extending above the mouths of the major hepatic veins [4–10]. However, the cases of intraluminal tumor removal from the right atrium with the help of these surgical techniques are quite rare in the world literature [9, 11–15].

We retrospectively evaluated our own experience in the surgical treatment of renal cell carcinoma spreading to the IVC and the right atrium with the use of the liver transplantation technique described by Ciancio et al. [10]. The features of this surgical technique, including access to the right atrium through the diaphragm from the abdominal cavity, and complications were analyzed. Particular attention was paid to the manual repositioning of the tumor thrombus apex below the diaphragm.

## 2. Materials and Methods

### 2.1. Characteristics of Patients and Tumors

Between 2002 and 2018, ninety-six nephrectomies with the removal of caval tumor thrombi in patients with renal cell carcinoma (RCC) were performed. The tumors spread above the diaphragm in 16 (16.7%) cases, including 5 (5.2%) patients with atrial thrombi.

The study included only those patients in whom the tumor thrombus apex was located in the lumen of the right atrium. This was confirmed by abdominal and chest multidetector computed tomography (MDCT) with contrast enhancement, as well as abdominal ultrasound examination. Ultrasonography demonstrated free flotation of tumor apex in the atrial lumen in all five patients.

The main characteristics of the patients and the tumors with atrial extension are presented in Table 1.

Among our patients, males predominated (3 : 2). The mean age of the patients was 58.8 ± 11.2 years (from 41 to 72 years). Their Eastern Cooperative Oncology Group (ECOG) score did not exceed 1 in 4 cases. A glomerular filtration rate (GFR) ranged from 36.8 to 76.2 ml/min (an average of 57.2 ± 16.0 ml/min), and a body mass index (BMI) was from 17.8 to 28.9 kg/m^2^ (an average of 24.8 ± 4.5 kg/m^2^). One patient had developed a significant body mass deficit.

Right-sided tumors were detected in 3 out of 5 patients. The average size of the tumors was 14.9 ± 9.4 cm. The histological examination confirmed RCC in all cases.

Two patients had synchronous lung metastases. In one patient, a giant kidney tumor spread into *m. psoas* and into the wall of the right common iliac artery.

Persistent swelling of the lower extremities was noted only in one patient with the right-sided tumor. Moderate symptoms of heart failure were observed in two patients. Their left ventricular ejection fraction was 44% and 48%. In the whole group, this parameter averaged 52 ± 5.7%.

**Figure 1:**
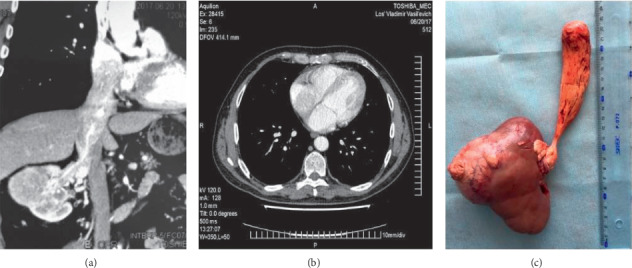
MDCT ((a) frontal reconstruction and (b) axial scan) and surgical specimen (c) of a patient with cavoatrial extension of RCC. The length of the atrial part of the tumor thrombus is 3.5 cm.

The length of the atrial part of the tumor thrombus ranged from 10 to 35 mm (20.0 ± 11.7 mm on average) and the width from 10 to 30 mm (14.8 ± 8.5 on average) (Figure 1). The average diameter of the tumor thrombus reached 32.6 ± 19.9 mm (13 to 63 mm). Presence of a blood clot, which was located below the tumor thrombus, was detected in 2 cases. A complete block of blood flow through the IVC by visual diagnosis occurred in three patients. Invasion of the intraluminal tumor into the caval wall was registered in 3 cases.

After surgical treatment, two patients with pulmonary metastases received systemic therapy with pazopanib. The mean follow-up period was 12.4 ± 3.6 months. The follow-up was performed by ultrasound examination every 3 months, as well as abdominal and chest MDCT every 6 months. Statistical analysis was carried out using standard descriptive statistical methods by means of “Statistica 8.0” software.

### 2.2. Surgical Technique

Patients with tumor extension to the right atrium were operated on with the help of the transplant technique for thrombectomy without extracorporeal circulation [10]. In all cases, “chevron” access was made. The duodenum was kocherized. Afterwards, liver mobilization was performed: the falciform, triangular, and coronal ligaments were transected. The piggy-back liver mobilization was carried out by section and ligation of the small hepatic veins draining into the anterior surface of the retrohepatic vena cava. We passed a tourniquet around the suprahepatic infradiaphragmatic IVC and proceeded to the surgical access to the supradiaphragmatic IVC and the right atrium without opening the pericardium.

**Figure 2:**
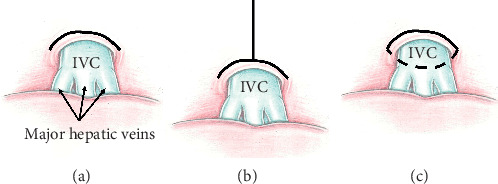
Types of diaphragmotomy: (a) transverse diaphragmotomy, (b) T-shaped diaphragmotomy, and (c) circular diaphragmotomy.

Initially, a transverse diaphragmotomy was performed, which included incision of the diaphragm parallel to the anterior semicircle of the inferior vena cava with the margin of 3–5 mm. This access was extended by a perpendicular longitudinal incision within 4-5 cm (T-shaped diaphragmotomy) (Figure 2). Then, a circular diaphragmotomy was performed, which involved total circular isolation of the IVC from the diaphragm, and the tourniquet was passed around the cavoatrial junction. The right semicircle of the supradiaphragmatic IVC was mobilized with extreme precautions. Surgical dissection in this area was made as close as possible to the caval surface to avoid the right phrenic nerve injury.

The right lobe of the liver was turned and moved to the medial side. Subsequently, the subhepatic and retrohepatic parts of the inferior vena cava were isolated. In cases of right-sided tumors, the renal artery was ligated through the interaorta-caval space. In patients with left-sided tumors, the renal artery was controlled posteriorly after mobilization and medial rotation of the kidney.

The next stage of the surgical intervention was the displacement of the tumor thrombus apex (“milking maneuver”). First, intraoperative transabdominal ultrasonography and palpation of the cavoatrial junction with the right atrium were performed. If the apical part of the intraluminal tumor extended to the right atrium by 10–15 mm, then it was covered by the thumb and index fingers of the left hand and displaced below the diaphragm afterwards. Further, a vascular clamp was placed directly above the upper end of the tumor, but below the cavoatrial junction. The pericardium was never opened in these patients. The tight fixation of the thrombus apex by fingers through the atrial walls was the main measure for the prevention of the intraluminal tumor fragmentation and pulmonary embolism.

In cases of large thrombi with the length of their intra-atrial part reaching 30–35 mm, transpericardial access to the right atrium was used. For this purpose, the pericardium was opened with a longitudinal incision for a length of 4.0–5.0 cm.

**Figure 3:**
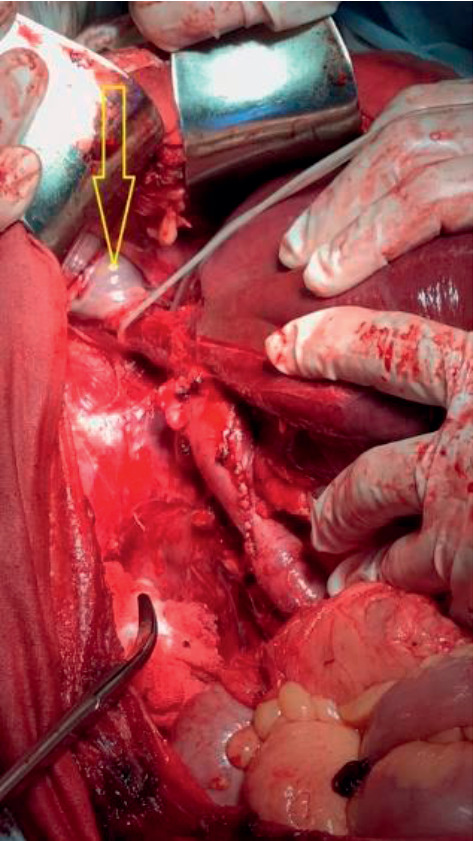
Transpericardial access to the right atrium (arrow) through the diaphragm from the abdominal cavity. Mobilization of the retrohepatic and supradiaphragmatic IVC with the liver piggy-back mobilization.

The incision of the pericardium continued around the anterior, right, and posterior sectors of the intrapericardial IVC. This maneuver provided wide access to the entire right atrium from the abdominal cavity (Figure 3). The displacement of the thrombus apex was carried out the same way as in the extrapericardial surgical access. Selection of the surgical access to the right atrium for performing the “milking maneuver” (either the extrapericardial access only, or the extended transpericardial access) was made in every patient individually based on palpation of the right atrium after a T-shaped and circular diaphragmotomy.

After the displacement of the thrombus below the diaphragm, a standard technique of three tourniquets was used to remove it. For control of bleeding from the hepatic veins, the Pringle maneuver was performed. The IVC was opened with a longitudinal incision from the level of the renal vein down to the lower edge of the major hepatic vein mouths. Immediately after evacuation of the thrombus, a tourniquet was placed on the IVC directly below the mouths of the major hepatic veins. The clamps were removed from the cavoatrial junction and the hepatoduodenal ligament.

## 3. Results

In all cases, the renal artery was ligated prior to thrombectomy. Since the right-sided tumors were present in most patients, control of the renal artery was fulfilled most often between the aorta and the IVC. However, in two cases, this stage of surgery was very difficult due to a large diameter and limited mobility of the inferior vena cava. Significant bleeding from the retroperitoneal venous collaterals, during mobilization and medial rotation of the kidney, took place in one of the patients with the left-sided tumor. The main characteristics of surgical interventions and complications are presented in Table 2.

Piggy-back liver mobilization was necessary in all five observations. At this stage of surgery, 4 to 10 short hepatic veins were transected (an average of 6.5 ± 2.6). During the access to the right atrium from the abdominal cavity, in most cases, only the anterior diaphragmatic veins were ligated at their confluence with the IVC (on average 2 ± 0.7 veins). In one case, the posterior diaphragmatic vein draining into the infra-diaphragmatic IVC was identified and transected.

Displacement of the thrombus apex below the diaphragm was successfully carried out in all 5 patients. In three patients with small atrial thrombi (up to 15 mm), it was sufficient to perform extrapericardial access to the right atrium for manual thrombus displacement. In two remaining patients with the thrombus atrial component length of 3.0 cm and 3.5 cm, control of the thrombus apex without opening the pericardium was impossible. Transpericardial access provided an opportunity for adequate palpation and fixation of the atrial component of the tumor thrombus by fingers. Displacement of the thrombus below the diaphragm in these patients passed without any significant difficulties. In all cases, the thrombus apex was moved below the cavoatrial junction and a clamp was placed at this level. The average clamping time of the supradiaphragmatic IVC was comparable to the average time of the Pringle maneuver, which was 5.6 ± 4.3 min. All patients were hemodynamically stable during this period. A moderate decrease in blood pressure was noted in 4 out of 5 patients (by 14.0 ± 8.9 mmHg on average).

Among intraoperative complications during the access to the right atrium, injuries to the main hepatic veins and the supradiaphragmatic IVC predominated (3 observations). However, these traumatic defects were small, not accompanied by any significant bleeding and easily repaired. Two patients had certain complications associated with liver mobilization—diaphragm and liver injuries, which also did not cause any serious problems. In one case, a giant kidney tumor spread to the right common iliac artery, which led to the need for its resection and placement of a vascular prosthesis.

Lateral resection of the suprarenal component of the IVC was performed in 3 patients due to invasion of the intraluminal tumor into the caval wall. The diameter of the IVC at the level of resection was at least 2/3 of the initial values.

In 2 cases, after evacuation of the tumor thrombus, a blood clot was removed from the infrarenal IVC and femoral veins using a Fogarty catheter. Recurrent thrombosis of the inferior vena cava caused by a blood clot was detected subsequently in one of these patients. Another patient had intestinal obstruction, which was managed conservatively. Specific complications associated with access to the right atrium from the abdominal cavity (paresis of the right phrenic nerve, pneumothorax, and mediastinitis) were not detected in any case.

The volume of intraoperative blood loss ranged from 2500 to 5600 ml and averaged 3540 ± 1248.2 ml. Most of the intraoperative blood loss was associated with damage to the massive venous collaterals during mobilization of the liver and IVC as well as with bleeding from the lumbar veins draining into the vascular isolation zone of the tumor thrombus. The average volume of transfused erythrocytes was 7.2 ± 2.7 units. The average operation time did not exceed 272 ± 36.3 min.

The length of ICU stay varied from 2 to 3 days (on average 2.4 ± 0.5 days), and the duration of hospital stay was 10–16 days (12.2 ± 2.3 days on average).

All the patients were still alive at the time of this work preparation. The median overall survival was 14 months. In three nonmetastatic patients, tumor progression was not detected during the entire follow-up period. One of two patients with pulmonary metastases receiving pazopanib in the postoperative period demonstrated bone and new pulmonary metastases, as well as local tumor recurrence in the wall of the inferior vena cava after 10 months. In another observation, there was no increase in size and number of pulmonary lesions within 14 months.

## 4. Discussion

The use of extracorporeal circulation and circulatory arrest during surgery of caval tumor thrombi extending above the diaphragm may be accompanied by serious complications (coagulopathy, neurological disorders, and multisystem failure) [2, 3]. Such approach significantly increases the trauma level and duration of the operation (the setup time for cardiopulmonary bypass is at least 30 minutes). On the other hand, one should take into account that the use of this technique requires median sternotomy, which also significantly increases the length of surgery and can result in serious postoperative complications. Therefore, many surgeons currently tend to search for alternative approaches for removing the supradiaphragmatic tumor thrombi without sternotomy, cardiopulmonary bypass, and circulatory arrest.

Surgical interventions without the use of extracorporeal circulation include three basic options for displacement of the thrombus apex downwards: the balloon technique, insertion of a finger into the right atrium, and external manual displacement (milking maneuver).

Control of the tumor thrombus apex with a balloon catheter wins by its simplicity and low invasiveness. Ability to remove the tumor masses from the lumen of the right atrium and inferior vena cava without complete liver mobilization, additional access to the mediastinum and opening the atrium, looks very tempting. However, the safety of this technique depends on several important conditions. First of all, the intraluminal tumor masses should not completely fill the caval lumen. The catheter should pass freely between the thrombus and caval wall. The dimensions of the thrombus atrial part should not be large. It is desirable for the thrombus to be of dense consistency. Finally, the tumor thrombus should not invade the caval wall.

The recent paper by Sobczyński et al. has demonstrated good results of using the balloon technique in four patients with cavoatrial tumor thrombi, which completely matches the abovementioned criteria [11]. However, one should take into account that atrial thrombi of a small diameter without signs of invasion into the caval wall are not very common. Considering our patients for possible thrombectomy with the balloon catheter, it should be noted that an average diameter of the tumor thrombus reached 32.6 ± 19.9 mm (13 to 63 mm); the atrial part of the tumor was 3.0 and 3.5 cm in two cases, and in three patients, there were the signs of caval wall invasion. Due to the abovementioned factors, the balloon catheter could be used potentially only in two out of five patients. However, from our point of view, displacement of the tumor thrombus apex with the balloon is a rather risky maneuver, which can be accompanied by thrombus fragmentation, pulmonary artery embolism, and incomplete removal of the intraluminal tumor in the case of its firm fixation to the caval wall. It is also necessary to consider the possibility of arrhythmia and acute hypotension due to the rapid decline of atrial volume during balloon inflation.

In 1989, Skinner et al. proposed displacement of the atrial thrombus with the help of the index finger inserted into the right atrium through a small incision on the beating heart [16]. The main advantages of this approach are direct control of the thrombus apex by tactile sensation of the surgeon, as well as the possibility of rapid displacement of the thrombus below the diaphragm or major hepatic vein mouths. In the study by Schneider et al., who used this surgical technique together with transesophageal echocardiography in 4 patients, the feasibility and safety of this procedure were demonstrated [9]. They noted that the risk of embolism and other complications during this maneuver was extremely low. Nevertheless, one should note the potential problems and limitations of this surgical technique, which include significant trauma (sternotomy or thoracolaparotomy and atriotomy), risk of bleeding, and air embolism. Although the authors have demonstrated minimal risk of thrombus fragmentation and tumor embolism, it still exists. It should also be borne in mind that displacement of the thrombus is carried out by pushing movements, so it is quite difficult to fix the thin and mobile thrombus apex in the lumen of the atrium beating by one finger.

Ciancio et al. introduced a liver transplantation technique for caval thrombectomy which included piggy-back liver mobilization with complete mobilization of the retrohepatic IVC, surgical access to the supradiaphragmatic IVC from the abdominal cavity, and external manual displacement of the thrombus below the diaphragm or major hepatic veins [10]. This surgical technique has become popular and now it is actively used to remove tumor thrombi reaching the retrohepatic and intrapericardial IVC [6, 15, 17, 18]. There are also rare reports on the use of this technique in patients with right atrial tumor thrombi. In particular, Cerwinka et al. described one patient with invasion of an atrial thrombus into the endocardium who was treated by this surgical method [14]. In 2010, Ciancio et al. presented successful results of the liver transplant technique in 7 patients with right atrial thrombi [13]. We also used this technique in our 5 patients with a modified surgical access to the right atrium.

The main advantages of thrombectomy with the help of the liver transplantation technique are the possibility of an easy access to the mediastinum through the diaphragm from the abdominal cavity and the rapid and safe displacement of the thrombus apex below the diaphragm or the mouths of the major hepatic veins. However, with respect to this method, there are several questions that need to be answered. In particular, what kinds of surgical accesses to the right atrium from the abdominal cavity are the most convenient? Are these approaches safe? How feasible and safe is the external displacement of the tumor thrombus from the atrium? What size of the tumor atrial part is acceptable for this technique?

In recent years, several reports have been published regarding the approaches to the intrapericardial part of the inferior vena cava and to the right atrium through the diaphragm from the abdominal cavity [5, 12, 15, 19–22]. All these approaches are divided into longitudinal, transverse, and circular (around the IVC) diaphragmotomy, or they are a combination of several incision types. Also, two other types of these approaches are distinguished as extrapericardial and transpericardial. Undoubtedly, extrapericardial approaches have certain priorities in terms of safety, since opening the pericardium during surgery can be accompanied by a decrease in cardiac output and, in the postoperative period, can lead to the development of constrictive or purulent pericarditis, as well as cardiac tamponade [23, 24].

In our study, in patients with tumor thrombi extending to the right atrium by 1.0 or 1.5 cm, the apex was easily palpated and shifted through the extrapericardial approach. However, in cases of atrial thrombi reaching 3.0 cm or more, it was impossible to perform external manual control of the thrombus apex without opening the pericardium.

**Figure 4:**
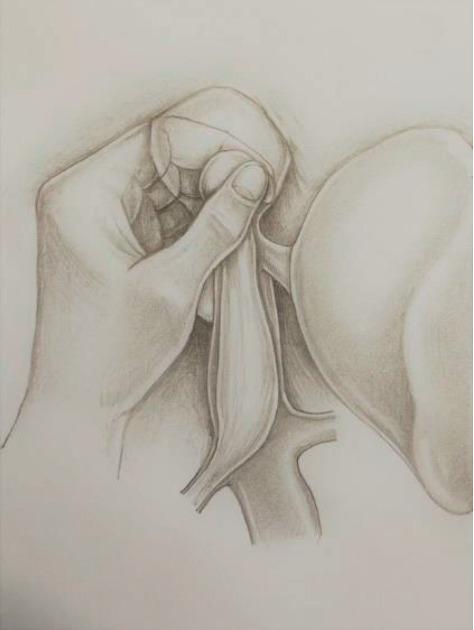
Performing “milking maneuver” in a patient with a large atrial tumor thrombus through the transpericardial access from the abdominal cavity.

Earlier we conducted an anatomical study devoted to the feasibility and safety of various surgical approaches to the supradiaphragmatic IVC from the abdominal cavity [25]. The best results were obtained in the case of extraperipercardial T-shaped diaphragmotomy (a combination of longitudinal and circular incisions of the diaphragm). In our clinical practice, we use this access most often, since it is convenient, not traumatic, and allows reliable manual control of the tumor thrombi located next to the cavoatrial junction. In situations where adequate control of the thrombus apex from T-shaped extrapericardial access was impossible, we performed longitudinal opening of the pericardium and continued incision around the anterior, right, and posterior sectors of the intrapericardial IVC. Such approach affords an opportunity for the successful performance of the “milking maneuver,” even when the length of the atrial part of the thrombus reaches 3.5 cm. One of the most important arguments in favor of the transpericardial approach is that the apex of a large thrombus, spreading into the lumen of the right atrium, extends not only upwards, but also to the left, along the course of the blood flow from the right atrium to the right ventricle. Therefore, for reliable fixation of the thrombus, the whole right atrium must be controlled by fingers (Figure 4).

Analysis of the presented surgical approaches to the right atrium, which were used in our clinical work, demonstrated their safety. The diaphragmatic veins drained mostly in the area of the anterior semicircle of the IVC. Only in one case, damage to the posterior surface of the IVC in the place of its confluence with the posterior diaphragmatic vein was identified. However, this damage was not accompanied by any significant bleeding and was easily repaired. In the postoperative period, we did not register any signs of mediastinitis, pericardial tamponade, or injury to the right phrenic nerve.

Regarding the stage of thrombus displacement from the right atrium, it should be noted that, in all cases, it was performed quickly and was not accompanied by any significant decrease of blood pressure, arrhythmia, or tumor embolism. We encountered moderate technical difficulties during performing this maneuver only in one patient with the largest atrial component of the thrombus (3.5 cm). The problem was associated with active movements of the tumor apex in the atrial lumen, which caused some issues with fixation and shifting of the tumor thrombus.

Considering a very small experience, it is currently difficult to estimate the maximal size of the atrial thrombus, for which the transplant technique for thrombectomy is safe to use. Nevertheless, as our study has demonstrated, these dimensions can be up to 3.5 cm. The liver transplantation technique during the removal of thrombi with the atrial component length up to 1.5 cm is safe and easily achievable. It actually does not differ from the technique used in patients with nonatrial supradiaphragmatic IVC thrombi.

The most serious problems of the transplantation technique in patients with cavoatrial thrombi are the inability to displace the thrombus apex below the diaphragm due to adhesions with the atrial endothelium, as well as hemodynamic instability in the moment of the cavoatrial junction clamping. In these situations, the safest option is to switch to cardiopulmonary bypass, which must be available to the surgical team. Although none of our patients needed extracorporeal circulation, we believe that these operations should be performed in a cardiac surgery operating unit.

Good visualization of the atrial part of the thrombus and identification of its mobility in the atrium are the most important factors for the success of the transplantation technique for thrombectomy. For these purposes, we used MDCT and transabdominal echocardiography before surgery, which were accurate in all patients. Nevertheless, it should be borne in mind that these methods have significant drawbacks. In particular, in patients with the thin cavoatrial thrombi, their atrial part is not clearly visible on MDCT due to high mobility of the intraluminal tumor. Performing transabdominal echocardiography may be difficult due to the constitutional features of patients or chest deformity. For intraoperative identification of the atrial tumor, we used transabdominal ultrasonography. This method is accurate, from our point of view, but not convenient enough. Undoubtedly, the transesophageal echography, which allows monitoring the position of the thrombus at all stages of the operation and makes surgical intervention safer, is the “gold standard” of intraoperative imaging of cavoatrial tumor thrombi.

It should be objectively recognized that surgical methods, including cardiopulmonary bypass with or without deep hypothermic circulatory arrest, are necessary for most atrial tumor thrombus removals. However, in distinct patients, this surgical problem can be solved with the help of the liver transplant technique for thrombectomy. From our point of view, while determining the indications for the use of this method, the leading role belongs to the experience of a surgeon in the operations on patients with retrohepatic and intrapericardial IVC thrombi. Another important aspect is careful selection of patients for performing such surgical interventions, which adhere to two main conditions: during ultrasonography, the thrombus apex should float freely in the atrial lumen and the patient should not have severe heart failure. However, further multicenter randomized studies are needed to determine the feasibility and safety of this surgical approach in comparison with other techniques.

## 5. Conclusion

Our work represents the initial experience in the use of the transplantation technique in distinct and well-selected patients with the right atrium tumor thrombi. The effectiveness of this approach needs further study.

## Figures and Tables

**Table 1:** Characteristics of patients and tumors with atrial extension.

	Case 1	Case 2	Case 3	Case 4	Case 5
Sex	M	F	M	F	M
Age	59	41	72	60	62
ECOG status	0	2	1	1	0
GFR (mL/min)	68.6	46.4	58.2	36.8	76.2
BMI (kg/m^2^)	24.3	17.8	28.9	25.3	28.6
Lesion side	Right	Right	Right	Left	Left
Tumor size (cm)	6.5	30	10	18	10.2
TNM	pT3cN0M1	pT4N0M1	pT3cN0M0	pT3cN0M0	pT3cN0M0
Metastasis localization	Lungs	Lungs	−	−	
Symptoms	Hematuria	Weight loss Weakness Pain	Hematuria Dyspnea Lower extremity swelling	Hematuria Weakness	Hematuria
Heart failure	−	−	+	+	−
LVEF (%)	55	57	48	44	56
Blood clot below tumor thrombus	−	+	+	−	−
Thrombus maximal diameter (mm)	32	38	63	17	13
Thrombus atrial part (mm)	Length-35 Width-30	Length-15 Width-12	Length-10 Width-10	Length-10 Width-12	Length-30 Width-10
Complete blood flow block in the IVC	+	+	+	−	−
Tumor invasion into the IVC wall	−	+	+	+	−
Follow-up (months)	14	15	9	8	16

**Table 2:** Characteristics of surgical procedures and complications.

	Case 1	Case 2	Case 3	Case 4	Case 5
Thrombus atrial part length (mm)	35	15	10	10	30
Piggy-back liver mobilization	+	+	+	+	+
Anterior phrenic veins transection	3	2	2	1	2
Posterior phrenic veins transection	1	−	−	−	−
Transpericardial approach	+	−	−	−	+
Successful external thrombus displacement	+	+	+	+	+
BP reduction during upper IVC clamping (mmHg)	20	−	20	10	20
Supradiaphragmatic IVC clamping time (min)	3	12	3	8	2
Major hepatic veins injury	+	−	−	+	−
Supradiaphragmatic IVC injury	−	−	+	−	−
Liver injury	−	−	+	−	−
Diaphragm injury	−	−	−	+	−
Other intraoperative complications	−	*A. iliaca* injury	−	−	−
Longitudinal IVC resection	−	+	+	+	−
Surgery time (min)	220	320	280	260	280
Blood loss volume (ml)	2500	5600	3800	2800	3000
Packed red blood cell transfusion volume (U)	5	11	9	5	6
Postoperative complications	−	IVC thrombosis	−	Intestinal obstruction	−

## Data Availability

The data used to support the findings of this study are available from the corresponding author upon request.
